# Psychological effects of trauma, negative and positive coping, resilience, and psychological distress among Chinese healthcare workers during the COVID-19 pandemic

**DOI:** 10.1016/j.xjmad.2023.100046

**Published:** 2024-01-06

**Authors:** Shujing Zhang, Daphne Y. Liu, Jinbing Bai, Jia-Chen Fu, Feng Jiang, Eric Nehl, Huanzhong Liu, Yanqun Liu, Chunhua Zhang, Yi-lang Tang, Nadine J. Kaslow

**Affiliations:** a Emory University School of Medicine, United States; b University of Denver, United States; c Emory University Nell Hodgson Woodruff School of Nursing, United States; d Academia Sinica Institute of Modern History, Taiwan; e Shanghai Jiao Tong University, China; f Emory University Rollins School of Public Health, United States; g Chaohu Hospital of Anhui Medical University, China; h Wuhan University School of Nursing, China; i Zhongnan Hospital of Wuhan University Nursing Department, China; j Atlanta Veterans Medical Center, Decatur, GA, United States

## Abstract

**Background:**

Limited data are available on risk (psychological effects of trauma, negative coping) and protective (resilience, positive coping) factors for psychological distress among Chinese healthcare workers (HCWs) during the pandemic. Thus, this study investigated the: (1) association between both the psychological effects of trauma and negative coping and psychological distress; and (2) moderating effects of resilience and positive coping on these associations.

**Methods:**

Participants (*n* = 196; *M*_age_ = 32.8; *SD*_age_ = 7.5; 77% female) from two hospitals in China completed self-report measures of the psychological effects of trauma (Impact of Event Scale-Revised), negative and positive coping (Simplified Coping Style Questionnaire), resilience (Connor Davidson Resilience Scale) and distress (Depression, Anxiety, and Stress Scale; Patient Health Questionnaire-9) in March 2022.

**Results:**

Results from this cross-sectional study revealed that HCWs who endorsed greater psychological effects of trauma had more psychological distress when they had lower levels of positive coping ((DASS-21 (*b* = −0.03, *SE* = 0.01, *p* = .007); PHQ-9 (*b* = −0.005, *SE* = 0.002, *p* = .015)). HCWs who endorsed more negative coping had more psychological distress when they were less resilient ((DASS-21 (*b* = −0.02, *SE* = 0.01, *p* = .035); PHQ-9 ((*b* = −0.01, *SE* = 0.002, *p* = .031)) and used less positive coping ((DASS-21 (*b* = −0.13, *SE* = 0.03, *p* < .001); PHQ-9 (*b* = −0.02, *SE* = 0.006, *p* < .001)).

**Conclusions:**

Psychological distress level was higher among HCWs who experienced more trauma or used negative coping strategies. They also had lower resilience and relied less on positive coping strategies. To help HCWs in China during public health crises, interventions must bolster their resilience and positive coping skills.

## Highlights

• Risk and protective factors for Chinese healthcare workers’ (HCWs) distress are key.
• Trauma exposure or negative coping lead to more psychological distress.
• Distress is lessened when HCWs in China are resilient or use positive coping.
• Culturally relevant interventions must bolster HCWs’ resilience and positive coping.

## 1 Introduction

Understanding and bolstering the psychological well-being of healthcare workers (HCWs, i.e., doctors, nurses, and other healthcare professionals working at inpatient and outpatient settings) during a global public health crisis, such as the COVID-19 pandemic, is imperative for optimizing patient care and the public health of our communities [2]. HCWs in China have been significantly impacted by the collective trauma of the pandemic as reflected in high prevalence rates of psychological distress (i.e., anxiety, depression, and insomnia) and burnout [9], [18], [22], [27]. What is more, studies have shown that these mental health symptoms may have a long-term impact on HCWs’ well-being, even years after the initial crisis has subsided [13].

Substantial data have been gathered on the patterns and risk factors for Chinese HCWs’ psychological distress. Previous studies showed the following non-exhaustive list of risk factors: long work hours, heavy workloads, severe shortages of medical resources, physical exhaustion, the risk of infection, and restrictive measures such as lockdown [22], [26]. Yet limited data are available on two variables that may be associated with psychological distress in this population, namely the psychological effects of trauma experiences and negative coping. Trauma relates to the subjective and objective aspects of a threatening experience that result in a maladaptive processing [30]. Numerous research findings revealed strong associations between the psychological effects of trauma and other forms of psychological distress. For instance, research highlighted that the COVID-19 pandemic triggered high levels of psychological distress among Chinese people [28]. Another important variable in the context of this research is negative coping. Coping is described as the thoughts and behaviors people use to handle the internal and external demands of stressful situations [21]. Based on an individual’s handling of the demands of a stressful situation, coping strategies are commonly identified as positive (or adaptive) or negative (or maladaptive), although the actual adaptiveness of these strategies often depends on situational or contextual factors [1]. Coping strategies have a close impact on psychological well-being. Specifically, negative coping, which refers to more passive approaches to coping such as denial, disengagement, intentional avoidance, and misuse of substances, has been shown to be associated with psychological distress and mental disorders including depression and anxiety [16], [32]. In a study of survivors after the Wenchuan earthquake in China, negative coping was a risk factor for developing PTSD after being rescued [16].

There is also limited information on factors that may promote psychological well-being among HCWs, particularly in the face of experiences of trauma and a tendency toward negative or maladaptive coping. Two potential protective factors worthy of attention are resilience and positive or adaptive coping [40]. Resilience refers to the capacity to cognitively and emotionally navigate stress in a manner that allows one to recover from difficult situations or crises (Block & Block, 1980; Block & Kremen, 1996). There is evidence that resilience predicted posttraumatic growth longitudinally among Chinese frontline HCWs during the pandemic [25]. Positive coping refers to adaptive psychological or behavioral responses that people utilize to alter the nature of the stressor itself or how they perceive the stressor [33]. Examples of positive coping relate to coping actively with setbacks by talking with others, seeking social support and advice from others, learning from others’ experiences, adopting a positive view of stress, self-regulating distressing emotions, finding alternative solutions, and participating in physical and recreational activities. Positive coping has been found to bolster the emotional and cognitive functioning of HCWs in China during the pandemic [3].

To address these gaps in the literature, this cross-sectional research was conducted by an interprofessional team of healthcare and mental health professionals from both the United States (US) and China. The overarching goal was to investigate risk and protective factors associated with the psychological distress of HCWs during the COVID-19 pandemic when the “zero COVID policy” in China was in place. The study had two specific aims. Aim 1 was to examine the association between the impact of the psychological effects of trauma and other forms of psychological distress and the moderating effects of the two protective factors (i.e., resilience and positive coping) on this association. We hypothesized that experiencing more psychological effects of trauma during the COVID-19 pandemic would be associated with more psychological distress. Additionally, we hypothesized that greater resilience and positive coping would buffer against the negative psychological effects of trauma on other forms of psychological distress. Aim 2 was to examine the association between negative coping and psychological distress and the moderating effects of the two protective factors on this link: resilience and positive coping. Negative coping was conceptualized as a predictor of psychological distress because of evidence of a positive association between the two in the Chinese population early in the pandemic [34], whereas positive coping was conceptualized as a moderator given that positive coping correlated with decreased psychological distress in the Chinese population at the outset of the pandemic and thus could serve as a protective factor to capitalize upon in interventions [42]. We hypothesized that negative coping would be positively associated with psychological distress. Similar to Aim 1, we also hypothesized that greater resilience and more positive coping would buffer against the negative impact of negative coping on psychological distress. We theorized that participants’ gender, age, education level, and experience working with COVID-19 patients could be associated with the psychological effects they experience related to trauma, coping style, and psychological distress [12], [42]; thus, these variables were evaluated as possible confounding variables. Information about additional relevant risk and protective factors can shed light on both intervention targets and larger-scale psychological wellness programming for HCWs in China.

## 2 Methods

### 2.1 Participants

The sample consisted of 196 HCWs recruited in March 2022 from two large, public hospitals located in East and Central China (Hospital A and Hospital B) who provided valid responses to study measures. Five HCWs were excluded due to unknown hospital site affiliation. Any HCW employed by either hospital was included in the study and there were no specific exclusion criterion. Voluntary sampling was utilized to recruit participants from the two hospitals, which are located in different regions in China. The survey was distributed to all staff at both sites online and HCWs were encouraged to respond (see more details in Procedures). A prior power analysis was performed using G*Power version 3.1.9.6 [10] to determine the adequate sample size for testing the study hypotheses. Results suggested that the minimum sample size to achieve 80% power and detect a small effect size (*f*^*2*^= 0.05; α = .05) for hierarchical linear regression analyses was *N* = 159. Therefore, the obtained sample size of N = 196 was sufficient to test the study hypotheses.

The demographic characteristics of the entire sample and the sample associated with each hospital site are shown in Table 1. The majority of the sample were women (153/196, 78.1%). The mean age [+ /-SD] was 32.8 [+ /− 7.5] years. Among the participants, 147 were nurses (75.0%), 37 were doctors (18.9%), and the remaining were a combination of medical intern students and others. In addition, more than two fifths (42.3%) of the participants reported experience directly working with COVID-19 patients. Most of the participants (83.6%) had a Bachelor’s degree. Of note, a medical degree in China is equivalent to a Bachelor’s degree in China.

**Table 1:** Participants’ Demographic and Work-Related Variables by Hospital Site.

	Full sample (*N*=196)	Hospital A (*n*=116)	Hospital B (*n*=80)	p*
Gender^a^				.841
Female	151 (77.0%)	88 (75.9%)	63 (78.8%)	
Male	44 (22.4%)	27 (23.3%)	17 (21.2%)	
Age	32.8 (SD=7.5)	33.1 (SD=8.0)	32.5 (SD=6.8)	.625
Highest education^b^				< .001
High school or below	1 (0.5%)	1 (0.9%)	0 (0%)	
Associate degree	13 (6.6%)	13 (11.2%)	0 (0%)	
Bachelor’s degree	163 (83.2%)	86 (74.1%)	77 (96.2%)	
Master’s degree	16 (8.2%)	13 (11.2%)	3 (3.8%)	
Doctorate degree	3 (1.5%)	3 (2.6%)	0 (0%)	
Profession				< .001
Doctor	38 (19.4%)	33 (28.4%)	5 (6.2%)	
Nurse	147 (75.0%)	73 (62.9%)	74 (92.5%)	
Medical intern student	5 (2.6%)	4 (3.4%)	1 (1.2%)	
Other	6 (3.1%)	6 (5.2%)	0 (0%)	
Has directly worked with COVID-19 patients (Yes)	81 (41.3%)	19 (16.4%)	62 (77.5%)	< .001
Workplace				.015
Primarily outpatient	8 (4.1%)	6 (5.2%)	2 (2.5%)	
Primarily inpatient	138 (70.4%)	73 (62.9%)	65 (81.2%)	
Mixed outpatient and inpatient	39 (19.9%)	31 (26.7%)	8 (10.0%)	
Other	11 (5.6%)	6 (5.2%)	5 (6.2%)	

*Note.* * *p* values were based on the Fisher’s exact test (FET) for categorical variables and independent sample t-test for continuous variables.

a The percentages for female and male do not add up to 100% because one participant selected “other” for gender, and the “other” category was excluded from FET analyses due to extremely low frequency of endorsement.

b Due to extremely low frequencies of certain categories of highest education, we combined the lowest two categories “high school or below” and “Associate degree” into one category and combined the highest two categories “Master’s degree” and “Doctorate degree” into one category for FET analyses.

### 2.2 Procedures

The research design, study procedures, and informed consent process were culturally appropriate for HCWs in China. The study was reviewed and approved by the Institutional Review Board (IRB; IRB # STUDY00003524) of a university in the Southeastern US with study support and cultural context letters provided by both study sites in China. In approving this study, the IRB carefully considered the China Data Privacy Law.

The study battery of questionnaires was built and delivered via *Wenjuanxing* (the questionnaire star), a widely used online questionnaire platform in China. Participants were approached and recruited via WeChat, a popular social media and messaging app in China. Participants were asked to complete the study battery, which included seven measures. Five of these measures served as the source of the data for this report. After acknowledging “yes” to participating in the online version of the informed consent process, participants were directed to complete all the study measures. Participants were compensated for their participation with a virtual gift card in RMB that was equivalent to $10 US dollars based on the currency exchange rate at the time the data were collected. This compensation rate was aligned with the norm in China for study participation.

### 2.3 Measures

All measures, which were in Simplified Chinese, were validated previously in Chinese populations to ensure they were culturally appropriate and relevant. Before the start of data collection, the study battery was pilot tested with adults in China to assure the appropriateness of the survey content and length.

#### 2.3.1 Outcome measures of psychological distress

We examined two constructs of psychological distress – internalizing symptoms and depressive symptoms. Internalizing symptoms broadly capture a variety of symptoms related to internalizing disorders (e.g., depressive and anxiety symptoms). Depressive symptoms represent a subset of internalizing symptoms more specific to depressive psychopathology. Testing our hypotheses using two separate measures of psychological distress with varying scopes of symptoms allows us to clarify whether our findings are specific to certain type of symptoms or measures.

Overall internalizing symptoms were measured using the Depression, Anxiety, and Stress Scale – Chinese version (DASS-21) [41], which served as one outcome measure of psychological distress. This 21-item self-report instrument, originally developed in the U.S. [24], focuses on negative emotional states of depression, anxiety, and stress in the past week. Participants responded to each item using a 4-point Likert scale, ranging from 0 (did not apply to me at all) to 3 (applied to me very much or most of the time). The total score was calculated by summing up all the items from each subscale and multiplying by two, with the total score ranging from 0 to 126. A higher total score indicates more severe depressive, anxious, and/or stress symptoms. A total score ≥ 60 is considered an indication for further psychiatric assessment [24]. Although the DASS-21 initially was developed to measure depression (DASS-depression), anxiety (DASS-anxiety), and stress (DASS-stress) separately, empirical evidence supports a single-factor model when using the DASS-21 on Chinese healthcare professionals [17]. Therefore, we adopted the DASS-21 total score in the analyses of this study, with excellent internal consistency reliability for the current sample (Cronbach’s alpha =.97).

The 9-item self-report Patient Health Questionnaire-9 (PHQ-9) also was used as an outcome measure to tap the severity of depressive symptoms more specifically [19]. Each of the PHQ-9 items is rated from 0 (not at all) to 3 (nearly every day). The total score ranges from 0 to 27, with 5–9 indicating mild depression, 10–14 reflecting moderate depression, 15–19 suggesting moderately severe depression, and over 20 denoting severe depression. The PHQ-9 is widely used in various countries, including China, and has excellent psychometric properties in the Chinese population [35]. The Cronbach’s alpha for the PHQ-9 in the current sample study was.94, indicating excellent internal consistency reliability.

#### 2.3.2 Risk predictor measures of psychological distress

The Impact of Event Scale-Revised (IES-R) was adopted to assess the psychological effects of trauma, one of the risk factors in this study [6]. The IES-R is designed to measure the subjective distress caused by chronic and acute traumatic events. Participants were asked to rate the 22 items based on their experiences over the prior seven days as related to the COVID-19 pandemic using a 5-point Likert scale ranging from 0 (not at all) to 4 (extremely). The total score was calculated by adding up all the items and ranged from 0 to 88. The higher the score, the more severe the distress symptoms. A score ≥ 33 suggests a possible diagnosis of PTSD [8]. In the current sample, the IES-R had excellent internal consistency reliability as evidenced by a Cronbach’s alpha of.97.

The second risk predictor was negative coping. One of the two subscales of the Simplified Coping Style Questionnaire (SCSQ) was used to assess negative or more passive forms of coping [39], namely the negative coping subscale (SCSQ-negative coping; 8 items). Examples of items on the negative coping subscale as translated from Chinese include: “accepting the reality because there is no other way out” and “trying to forget the whole situation.” Each item of the SCSQ is scored on a 4-point Likert scale (0 = never used to 3 = often used), with a higher score indicating more utilization of the relevant coping strategies. The negative coping style subscale of the SCSQ in this study had excellent internal consistency reliability indicated by a Cronbach alpha of.87, consistent with data from other studies.

#### 2.3.3 Moderators: protective factors

The Connor Davidson Resilience Scale (CD-RISC) [7] was employed to assess the first protective factor, resilience. The CD-RISC contains 25 items that respondents rate using a 5-point Likert scale ranging from 0 (never) to 4 (always). The total score ranges from 0 to 100; higher scores suggesting greater resilience. In the current sample, the CD-RISC demonstrated excellent internal consistency reliability (Cronbach’s alpha =.98).

The positive coping subscale of the aforementioned SCSQ [39] measured the second protective factor, positive or more active forms of coping. Example items as translated from Chinese include “asking advice from relatives, friends, or coworkers” and “seeking hobbies and actively participating in cultural and sports activities.” This subscale includes 12 items scored as above. This subscale had excellent internal consistency reliability as indicated by a Cronbach’s alpha of.95, consistent with data from other investigations.

### 2.4 Data analysis

All analyses were conducted using R software (v. 4.1.2) [29]. We first obtained description statistics and correlations among study variables using Pearson’s correlation coefficients. Then, we examined differences in demographic and work-related variables between participants from different hospital sites (see Table 1) using independent samples t-test for continuous variables or Fisher’s exact test for categorical variables. Prior to conducting the subsequent analyses, all continuous variables were centered before being entered into the regression models. Initially, we included participants’ gender, age, education, level, and experience working with COVID-19 patients, and hospital site as covariates, but only age and education level exhibited significant associations and were retained for final analyses.

To address Aim 1, we conducted hierarchical linear regression analyses for the IES-R with the DASS-21 and the PHQ-9 as the psychological distress outcome measures, respectively. For each outcome measure, we conducted separate sets of analyses for each hypothesized moderator (i.e., protective factors: resilience and positive coping), totaling four sets of analyses. In each set of analyses, we first entered the psychological effects of trauma and the hypothesized moderator as predictors to obtain their main effects in predicting the outcome measure (Step 1), and subsequently entered the interaction between the impact of the psychological effects of trauma and hypothesized moderator as a predictor to test the moderating effect (Step 2). Significant interactions were followed up with simple slope analyses examining the effect of the predictor at high (+1 SD) and low (−1 SD) levels of the moderator. Of note, as many researchers have used the DASS-21 subscales instead of the DASS-21 total score, we repeated these analyses and those detailed below for Aim 2 using the DASS-21 subscale scores as the outcome variables to test whether the findings would be consistent.

For Aim 2, we conducted hierarchical linear regressions to predict each psychological distress outcome (DASS-21 or PHQ-9) based on the participants’ SCSQ-negative coping score. For each outcome measure, we conducted analyses for each hypothesized moderator (i.e., resilience and positive coping). Like Aim 1, within each set of analyses, we first entered negative coping and the hypothesized moderator as predictors to obtain their main effects (Step 1) and then entered the interaction between negative coping and the hypothesized predictor to test the moderating effect (Step 2). Significant interactions were followed up with simple slope analyses examining the effect of the predictor at high (+1 SD) and low (−1 SD) levels of the moderator.

## 3 Results

### 3.1 Basic features of participants

Table 1 shows that the participants from the two hospital sites differed significantly in education level, profession, workplace, and experience working directly with COVID-19 patients, but they did not differ in gender or age. Table 2 presents descriptive statistics and zero-order correlations of all study variables. Internalizing symptoms and depressive symptoms were positively correlated with psychological effects of trauma and negative coping and negatively correlated with resilience, but were not significantly correlated with positive coping. Negative and positive coping were positively correlated with each other, which while somewhat counterintuitive is consistent with findings from previous studies (e.g., *r* = .43) [23] and likely reflects the fact that both subscales relate to a person’s general tendency to utilize any coping strategy when exposed to a stressful event.

**Table 2:** Means (M), Standard Deviations (SD), and Zero-Order Correlations of Study Variables.

	*M* (*SD*)	2	3	4	5	6
1. Internalizing symptoms (DASS-21)	22.0 (23.5)	.77 * **	.64 * **	.35 * **	-.23 * *	-.14
2. Depressive symptoms (PHQ-9)	6.42 (5.37)	-	.66 * **	.35 * **	-.16 *	-.07
3. Psychological effects of trauma (IES-R)	28.9 (16.8)		-	.43 * **	-.14	-.02
4. Negative coping (SCSQ negative coping subscale)	10.8 (5.4)			-	.23 * *	.48 * **
5. Resilience (CD-RISC)	60.3 (20.0)				-	.63 * **
6. Positive coping (SCSQ positive coping subscale)	22.7 (8.5)					-

*Note.* CD-RISC = the Connor Davidson Resilience Scale; DASS-21 = the Depression Anxiety Stress Scales – Chinese version; PHQ-9 = the Patient Health Questionnaire; SCSQ = the Simplified Coping Style Questionnaire. All 196 participants had valid data for all six study variables reported in this table.

* *p* < .05, * * *p* < .01, * ** *p* < .001

For internalizing symptoms (DASS-21), 8.0% of participants scored above the clinical cutoff indicating severe psychological distress (≥60; [24]. Regarding depressive symptoms (PHQ-9), 22.4% of participants’ scores indicated at least moderate depressive symptoms (≥10; [19]. Additionally, 41.3% of participants reported symptoms that likely indicated the presence of PTSD based on the cutoff score of the IES-R (≥3; [8].

### 3.2 Primary analyses

#### 3.2.1 Impact of the psychological effects of trauma on psychological distress (Aim 1)

We examined the association between the psychological effects of trauma (IES-R) and psychological distress (DASS-21 or PHQ-9 total scores) as well as moderators (CD-RISC and SCSQ positive coping domain scores) of the association. Results for models including resilience (Panel A) and positive coping (Panel B) as potential moderators are presented in Table 3.

**Table 3:** Hierarchical Linear Regressions Testing Moderators of the Associations of Psychological Effects of Trauma with Internalizing Symptoms (DASS-21) and Depressive Symptoms (PHQ-9).

	DASS-21						PHQ-9					
	Step 1			Step 2			Step 1			Step 2		
Predictors	*b*	*SE*	*p*	*b*	*SE*	*P*	*b*	*SE*	*p*	*b*	*SE*	*p*
Panel A: Resilience as Moderator												
Psychological effects of trauma	0.91	0.08	< .001 * **	0.85	0.53	.112	0.21	0.02	< .001 * **	-0.07	0.12	.546
Resilience	-0.15	0.07	0.032 *	0.05	0.23	.822	-0.02	0.02	.118	0.07	0.05	.138
Psychological effects of trauma × Resilience				-0.002	0.004	.516				-0.000	0.001	.456
Panel B: Positive Coping as Moderator												
Psychological effects of trauma	0.93	0.08	< .001 * **	0.91	0.48	.061	0.22	0.02	< .001 * **	0.05	0.11	.631
Positive coping	-0.32	0.16	.036 *	-0.69	0.56	.220	-0.04	0.03	.220	-0.18	0.12	.145
Psychological effects of trauma × Positive coping				-0.03	0.01	.007 * *				-0.005	0.002	.015 *

*Note.* Unstandardized regression coefficients are presented. Participants’ age, gender, highest education, site, and experience working with COVID-19 patients were initially included as covariates. Age and highest education emerged as significant covariates in some models in Step 1 and thus were included as covariates at appropriate levels (controlled as main effects in Step 1 and at the interaction level in Step 2) in the final models for all analyses. Results included in this table represent models controlling for age and highest education (reference level = Associate degree or below). Because five participants did not report age, they were not included in our main analyses and resulted in an empirical sample size of 191 for these analyses. DASS-21 = the Depression Anxiety Stress Scales – Chinese version; PHQ-9 = the Patient Health Questionnaire.

* *p* < .050, * * *p* < .010, * ** *p* < .001

**Fig. 1:**
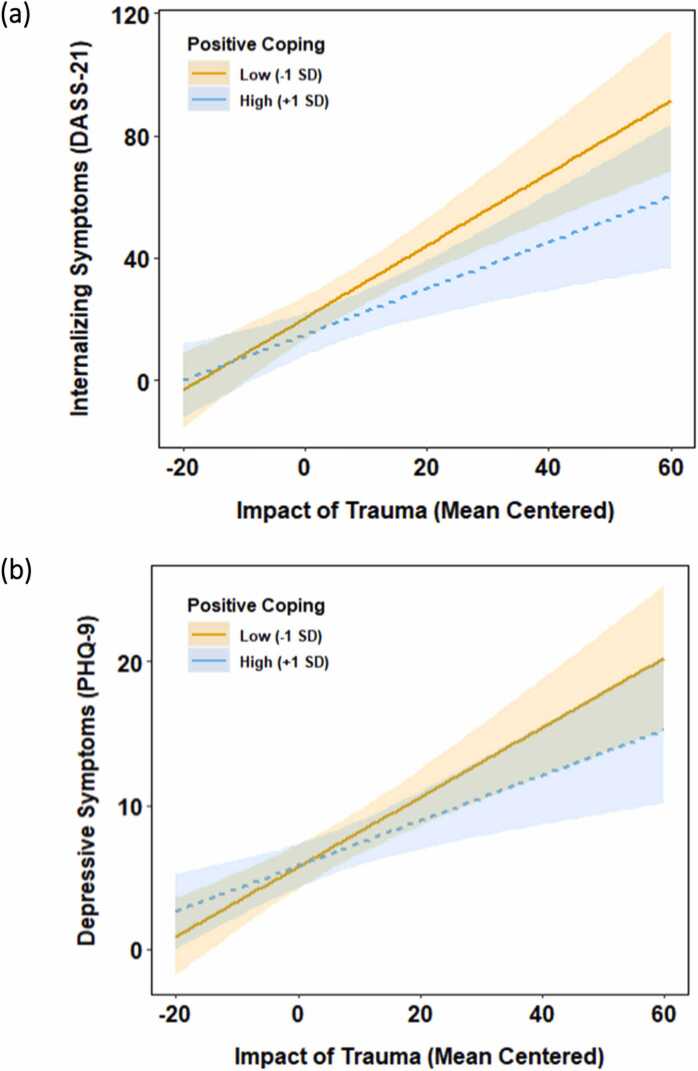
*Psychological Distress as a Function of the Psychological Effects of Trauma, as Moderated by Positive Coping, Note.* DASS-21 = the Depression Anxiety Stress Scales; PHQ-9 = the Patient Health Questionnaire. Shaded regions delineate the 95% confidence bands for the simple slopes.

For models using DASS-21 scores as an outcome, models for both resilience and positive coping revealed a main effect of the impact of the psychological effects of trauma (*p’*s < .001), with higher psychological effects of trauma predicting higher DASS-21 scores. Resilience showed a main effect in predicting DASS-21 scores (*b* = −0.15, *SE* = 0.07, *p* = .032), but its interaction with the psychological effects of trauma was not significant (*b* = −0.002, *SE* = 0.004, *p* = .516). Positive coping showed a main effect (*b* = −0.32, *SE* = 0.16, *p =* .036), and it interacted with the impact of psychological effects of trauma in predicting DASS-21 scores (*b* = −0.03, *SE* = 0.01, *p* = .007). Simple slope analyses showed that higher psychological effects of trauma predicted higher DASS-21 scores among participants with all levels of positive coping, but this association was weaker for those who reported high (*b* = 0.75, *SE* = 0.21, *p* < .001) versus low (*b* = 1.18, *SE* = 0.20, *p* < .001) levels of positive coping (Fig. 1a).

Models predicting PHQ-9 scores showed a similar pattern as those predicting DASS-21 (Table 3). The main effect of the psychological effects of trauma was significant in both models (*p*’s < .001). Resilience did not show a main effect (*b* = −0.02, *SE* = 0.02, *p* = .118), nor did it interact with the psychological effects of trauma in predicting PHQ-9 scores (*b* = −0.000, *SE* = 0.001, *p* = .456). Positive coping did not show a main effect (*b* = −0.04, *SE* = 0.03, *p* = .220), but it interacted with the psychological effects of trauma in predicting PHQ-9 scores (*b* = −0.005, *SE* = 0.002, *p* = .015). Simple slope analyses revealed that higher psychological effects of trauma predicted higher PHQ-9 scores at all levels of positive coping, but this association was weaker among participants with high (*b* = 0.16, *SE* = 0.05, *p* < .001) versus low (*b* = 0.24, *SE* = 0.04, *p* < .001) levels of positive coping (Fig. 1b).

#### 3.2.2 Impact of negative coping style on psychological distress (Aim 2)

**Fig. 2:**
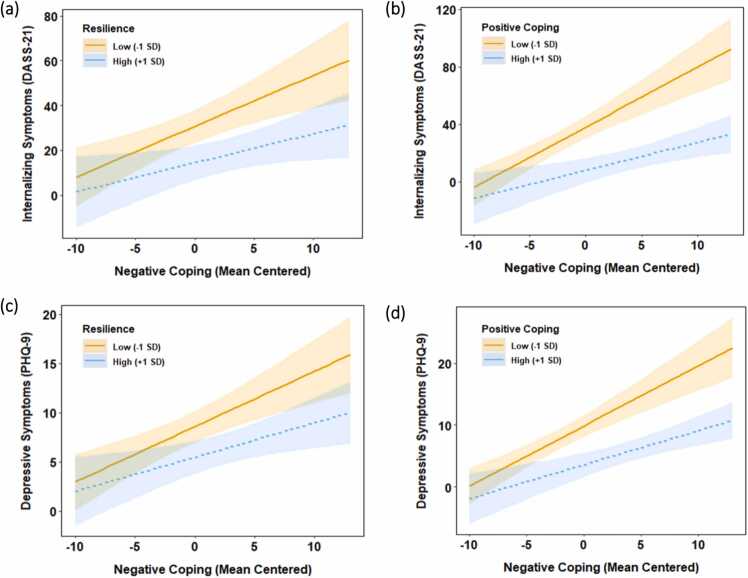
*Psychological Distress as a Function of Negative Coping, as Moderated by Resilience (a and c) and Positive Coping (b and d).Note.* DASS-21 = the Depression Anxiety Stress Scales; PHQ-9 = the Patient Health Questionnaire. Shaded regions delineate the 95% confidence bands for the simple slopes.

We examined the association between negative coping (SCSQ negative coping) and psychological distress (DASS-21, PHQ-9) and moderators (CD-RISC, SCSQ positive coping) of this link. Results for the moderating effects of resilience (Panel A) and positive coping (Panel B) are summarized in Table 4. For models predicting DASS-21 scores, both models showed a main effect of negative coping; more negative coping predicted higher internalizing symptoms (*p′*s < .001). Results also revealed a main effect of resilience (*b* = −0.38, *SE* = 0.08, *p* < .001) and an interaction between resilience and negative coping in predicting DASS-21 scores (*b* = −0.02, *SE* = 0.01, *p* = .035). Simple slope analyses showed that negative coping was positively associated with DASS-21 scores, but this association was weaker for those who reported high (*b* = 1.30, *SE* = 0.60, *p* = .029) versus low (*b* = 2.27, *SE* = 0.60, *p* < .001) levels of resilience (Fig. 2a). Similarly, positive coping showed a main effect (*b* = - 1.09, *SE* = 0.21, *p* < .001) and interacted with negative coping in predicting DASS-21 scores (*b* = −0.13, *SE* = 0.03, *p* < .001). Simple slope analyses showed that more negative coping predicted higher DASS-21 scores internalizing symptoms, but this association was weaker among participants who scored high (*b* = 1.95, *SE* = 0.61, *p* = .002) versus low (*b* = 4.20, *SE* = 0.68, *p* < .001) on positive coping (Fig. 2b).

**Table 4:** Hierarchical Linear Regressions Testing Moderators of the Associations of Negative Coping with Internalizing Symptoms (DASS-21) and Depressive Symptoms (PHQ-9).

	DASS-21						PHQ-9					
	Step 1			Step 2			Step 1			Step 2		
Predictors	*b*	*SE*	*p*	*b*	*SE*	*P*	*b*	*SE*	*p*	*b*	*SE*	*p*
Panel A: Resilience as Moderator												
Negative coping	1.83	0.29	< .001 * **	-0.08	1.12	.940	0.40	0.07	< .001 * **	-0.39	0.24	.115
Resilience	-0.38	0.08	< .001 * **	0.04	0.25	.888	-0.08	0.02	< .001 * **	0.04	0.05	.465
Negative coping × Resilience				-0.02	0.01	.035 *				-0.01	0.002	.031 *
Panel B: Positive Coping as Moderator												
Negative coping	2.34	0.32	< .001 * **	2.81	1.35	.040 *	0.48	0.07	< .001 * **	0.38	0.31	.211
Positive coping	-1.09	0.21	< .001 * **	-2.20	0.84	.010 *	-0.20	0.05	< .001 * **	-0.47	0.19	.014 *
Negative coping × Positive coping				-0.13	0.03	< .001 * **				-0.02	0.006	< .001 * **

*Note.* Unstandardized regression coefficients are presented. Participants’ age, gender, highest education, site, and experience working with COVID-19 patients were initially included as covariates. Only age and highest education emerged as significant covariates in some models in Step 1 and thus were included as covariates at appropriate levels (controlled as main effects in Step 1 and at the interaction level in Step 2) in the final models for all analyses. Results included in this table represent models controlling for age and highest education (reference level = Associate degree or below). Because five participants did not report age, they were not included in our main analyses and resulted in an empirical sample size of 191 for these analyses. DASS-21 = the Depression Anxiety Stress Scales – Chinese version; PHQ-9 = the Patient Health Questionnaire.

* *p* < .050, * * *p* < .010, * ** *p* < .001

Results for PHQ-9 showed similar patterns as those for DASS-21 (Table 4). The main effect of negative coping was significant in both models (*p′s* < .001). Resilience showed a main effect (*b* = −0.08, *SE* = 0.02, *p* < .001), and interacted with negative coping in predicting PHQ-9 scores (*b* = −0.01, *SE* = 0.002, *p* = .031). Simple slope analyses suggested that negative coping was associated with higher PHQ-9 scores all levels of resilience, but this association was weaker among those with high (*b* = 0.35, *SE* = 0.13, *p* = .008) versus low (*b* = 0.56, *SE* = 0.13, *p* < .001) levels of resilience (Fig. 2c). Similarly, positive coping showed a main effect (*b* = −0.20, *SE* = 0.05, *p* < .001), and an interaction with negative coping in predicting PHQ-9 scores (*b* = −0.02, *SE* = 0.006, *p* < .001). Simple slope analyses showed that negative coping predicted higher PHQ-9 scores, but this association was weaker among participants who scored high (*b* = 0.55, *SE* = 0.14, *p* < .001) versus low (*b* = 0.98, *SE* = 0.10, *p* < .001) on positive coping (Fig. 2d).

#### 3.2.3 Follow-Up analyses: DASS-21 subscales as the outcome variable

When replicating the analyses using each DASS-21 subscale, the result patterns remained almost identical for the depression, anxiety, and stress subscales. Since subscale score results were consistent with those found with DASS-21 total score as the outcome, they are reported in online supplemental materials. This is not surprising as this result is similar to those from a comparable sample [17] in which these subscales were correlated, *r*’s = .91–.92.

## 4 Discussion

Since the onset of the COVID-19 pandemic, numerous studies showed that psychological symptoms in HCWs in China have been prevalent and there is an urgent need for interventions to reduce this distress [36], [38]. Unfortunately, however, mental health professionals in China faced overwhelming challenges, especially at the initial stage of the pandemic, due to the lack of relevant psychological intervention guidelines, insufficient mental health resources, and inadequate training to provide mental health services in infectious units and hospitals. Moreover, even when mental health services were made available, HCWs in China often are reluctant to seek professional psychological support despite facing such unprecedented stress and demands during a public health crisis such as the pandemic [5], [37]. Taken together, these high levels of distress, challenges in implementing psychological wellness services throughout the country in a timely fashion, and reluctance to seek services may mean that many HCWs are not performing optimally. This has led to calls for international collaborations to better understand and address the mental health challenges faced over time by the HCWs in China [37].

This interdisciplinary global mental health research effort is one response to these calls. Through this cross-nation research collaboration, findings from this cross-sectional study highlighted the impact of both the psychological effects of trauma and negative coping on the psychological distress of HCWs in China during the latter stage of the pandemic. Our results are most novel in their underscoring of the critical role of both resilience and positive coping in explaining the strength of the association between the psychological effects of trauma and negative coping on the one hand and psychological distress on the other hand. As we hypothesized, HCWs who endorsed greater psychological effects of trauma during the pandemic had poorer psychological well-being and this association was moderated by positive coping, such that those HCWs in China who experienced a high psychological impact of trauma were most likely to have significant psychological distress when they relied less on positive coping. In addition, as predicted, HCWs who tended to rely more heavily on a negative coping style had more psychological distress, a finding that was particularly true for individuals with low levels of positive coping and/or low levels of resilience. These findings are consistent with data from other countries showing that resilience and effective coping strategies along with strong social support preserved the psychological well-being of HCWs throughout the pandemic [14], [20], whereas job-related stress, maladaptive coping, and poor support were associated with high levels of burnout [31]. In addition, findings from the current investigation are aligned with data showing that higher levels of resilience and positive coping strategies enhanced personal growth and more specifically, posttraumatic growth, for both HCWs and non-healthcare workers during the pandemic [11]. For frontline HCWs, such as those in the current investigation, this is particularly true when they receive psychological interventions or trainings within their workplace that empower them to generate a positive reappraisal of trauma events [11]. Taken together, our findings and related research highlight the value of offering interventions or providing workshops that enhance resilience and positive coping and guide HCWs in China in reframing crises as opportunities that can promote personal growth. Unfortunately, despite national recommendations highlighting the value of psychological interventions for HCWs, our sample of HCWs had limited access to such interventions during the pandemic.

### 4.1 Study limitations

While this investigation yielded important findings, some limitations need to be considered. The first set of limitations pertain to aspects of the study design. The use of voluntary sampling, despite its advantages, is susceptible to bias and raises questions about the representativeness of the sample. This study utilized self-report measures only and thus recall bias cannot be ruled out. In addition, several of the measures used, such as IES-R and CDRS, had clinical cut-scores from Western samples. The relevance of these scores to the current sample is unknown. Further, we did not include all relevant psychological distress outcomes (e.g., substance misuse, sleep disorder) nor did we compare the findings of our HCW sample to another sample, both of which would have strengthened the study design [4], [15]. Moreover, since a cross-sectional design was employed, we cannot draw any causal conclusions. The second set of limitations relate to the generalizability of the findings given the relatively small and non-representative nature of the sample. More specifically, the applicability of the results to HCWs outside of major cities and public hospital settings is unclear. In addition, given that the study was conducted in a specific phase of the pandemic, namely, after the early phases of the pandemic yet at a time during which the “Zero COVID policy” was still in place, it is questionable if the findings are applicable to other phases of a public health crisis.

### 4.2 Concluding comments

Despite these limitations, this project has shed light on two major protective factors that can be capitalized upon in creating, implementing, and disseminating wellness interventions for HCWs in China. It will be important to create culturally and contextually relevant wellness programs that bolster resilience and positive coping in a manner that mitigates burnout and empowers HCWs to function optimally and flourish including during times of public health crisis. Such programs must be able to be mobilized efficiently and effectively and be responsive to the unique need of diverse groups of HCWs in China. The emerging vibrant therapeutic culture in China combined with pandemic-related programming already in place provides a solid foundation for such innovative direction.

#### Funding

The funding we received in support of this study is Emory University, Emory Global Health Institute, Atlanta, Georgia. There is no grant number.

## Declaration of Competing Interest

The authors declare that they have no known competing financial interests or personal relationships that could have appeared to influence the work reported in this paper. None of the authors have any conflicts of interest to report. This research was supported by a grant from the Emory University Global Health Institute. The data that support the findings of this study are available from the corresponding author upon reasonable request.
